# An Exploration of Maternal Health Care Providers’ Perspectives on Respectful Maternity Care in the United States: A Scoping Review Protocol

**DOI:** 10.1177/24731242251384093

**Published:** 2025-10-01

**Authors:** Celestine Yayra Ofori-Parku

**Affiliations:** Department of Family Health Care Nursing, School of Nursing, University of California San Francisco, San Francisco, California, USA.

**Keywords:** respectful maternity care, maternal health care providers, mistreatment, maternal outcomes, United States, scoping review protocol

## Abstract

**Background:**

Maternal mortality and morbidity rates in the United States among racially minoritized populations have continued to worsen over the past decade. Reviews have examined the maternity care experiences and outcomes of Black individuals in the United States. However, few reviews have examined maternal health care providers’ experiences in the United States.

**Purpose:**

To comprehensively assess the current evidence and knowledge gaps related to maternal health care providers’ perspectives on providing respectful maternity care to Black patients.

**Method:**

The methodology is in accordance with the Preferred Reporting Items for Systematic Review and Meta-Analysis extension for Scoping Reviews guidelines, and the Joanna Briggs Institute enhanced scoping review framework. A literature search using medical subject headings on the terms “Maternity care providers,” “Perspective,” “Respectful maternity care,” “Person-centered maternity,” “Compassionate maternity care” together with BOOLEAN operators (“AND”/“OR”) will be conducted via PubMed, Embase, Web of Science, and CINAHL. The search will be limited to studies conducted in the United States, in English, and from 2013 to 2024. A Gray literature search will be conducted. Retrieved articles will be imported into Zotero to remove duplicates and imported to Covidence. Studies will be appraised using the Mixed Methods Appraisal Tool. A random-effects meta-analysis will be conducted based on the homogeneity of the included studies. If quantitative synthesis or meta-analysis is not appropriate due to the heterogeneity of the included studies, a combination of narrative and thematic synthesis methodology will be employed.

**Ethics and Dissemination:**

Ethics approval is not required for the scoping review, and these findings will be submitted for open-access publication in peer-reviewed journals and presented at scientific conferences.

**Discussion:**

The findings from this scoping review will help provide a comprehensive summary of clinicians’ perspectives on delivering facility-based maternity care free of discrimination and maltreatment, which can significantly inform policies to enhance quality and equitable maternity care services.

## Introduction

Maternal death or mortality (refers to “the death of a woman while pregnant or within 42 days of termination of pregnancy, irrespective of the duration and site of the pregnancy, from any cause related to or aggravated by the pregnancy or its management but not from unintentional or incidental causes”)^1^ is a growing public health problem globally. Thousands of women die or suffer pregnancy-related and delivery complications each year across the globe. For example, in 2020, approximately 800 women died daily from pregnancy and childbirth-related causes, for about 287,000 women at the end of 2020.^2^ Despite spending twice as much as average on health care as any other country in the global North, the U.S. maternal mortality rate is higher than any other country in the global North.^3,4^ About 1205 women died in 2021 in the United States from maternal causes, representing a 40% increase from 2020.^4^

Although the most tragic outcome for mothers is death, severe maternal morbidity (SMM) affects roughly 55,000 women annually in the United States and is 100 times more common than death.^5,6^ SMM refers to near misses or unforeseen effects of labor and delivery such as acute myocardial infarction, blood product transfusions, aneurysm, acute renal failure, adult respiratory distress syndrome, hysterectomy, and amniotic fluid/air embolism, which have immediate severe or long-term effects on the health of a birthing mother.^7^ According to the Centers for Disease Control and Prevention, a significant racial disparity exists in the maternal mortality and morbidity crisis. Black women bear the highest burden of this maternal mortality and morbidity.^8,9^ Black women are three to four times more likely to die from pregnancy-related causes than White women. Black birthing women not only deal with morbidity and mortality challenges every pregnant woman faces but they also grapple with the experience of racism and discrimination associated with being Black.^10–12^ These injustices stem from gender and racial inequalities, which impede access to culturally appropriate, inclusive health care for Black women during prepregnancy, pregnancy, and postpartum periods.^13^ Black women endure some of the highest rates of microaggression in health care settings.^14,15^

The mistreatment of Black women during maternity care is tied to the history of enslavement and medical racism in the United States.^16^ During chattel slavery, enslaved African women experienced a lack of reproductive autonomy and medical experimentation without consent or proper analgesics.^17^ In addition, medical racism speaks to how patients’ race influences medical professionals’ perceptions, diagnostic decisions, and treatments, placing patients at risk of harm.^17,18^ The differential treatment of Black and other racially minoritized pregnant and birthing people is termed obstetric racism.^17,18^ The concept of obstetric racism lies at the intersection of obstetric violence and medical racism, highlighting the forms of violence and abuse perpetuated by racism that Black women experience while seeking maternal health care.

Although the majority of prenatal care and births occur in facilities (mainly hospitals, clinics, and, to a lesser extent, birth centers),^19,20^ Black women tend to receive lower quality maternity care and have poor health outcomes than their White counterparts.^2,10,11,21,22^ Providers’ intentional or unintentional mistreatment^23,24^ of pregnant and birthing women during the pregnancy and postpartum period constitutes a violation of their human rights and signifies poor-quality care.^25^ Respectful maternity care refers to the provision of evidence-based and culturally sensitive care that upholds the rights of birthing individuals while minimizing mistreatment, discrimination, and obstetric violence.^26–29^ However, although women have a fundamental right to high-quality and equitable care, the opposite is prevailing in many facility-based maternity care settings, leading to adverse maternal outcomes for Black women.^26,30–32^ Despite the growing body of work in the field of Black maternal health care experiences, no scoping review has comprehensively evaluated maternal health care clinicians’ perspectives about provider-level, health-facility, and health system-level barriers to providing respectful maternity care in the United States.

### Objectives

This review aims to evaluate the current evidence and knowledge gaps related to maternal health care providers’ perspectives on providing respectful maternity care to Black patients in health care facilities (i.e., hospitals, clinics, and birth centers). This scoping review only focuses on the maternal health care providers’ perspectives of respectful maternity care during pregnancy, delivery, and the postpartum period.

## Methods

This scoping review will be conducted in accordance with the guidelines and checklist provided by the Preferred Reporting Items for Systematic Review and Meta-Analysis extension for Scoping Reviews (PRISMA-ScR).^33^ The review procedures will also be guided by the Joanna Briggs Institute enhanced scoping review framework.^34^

### Eligibility criteria

Supplementary Appendix A1 shows the inclusion and exclusion criteria that are developed using the participants, concepts, context, and types of sources of evidence (PCC framework).

### Data sources and search strategy

A literature search will be conducted in the following four databases: PubMed, Embase, Web of Science, and CINAHL. The search will be limited to studies conducted in the United States, in the English language, and from 2013 to 2024. This period was chosen because there has been an increase in proposed policies to address maternal health and racial disparities in the United States between 2010 and 2022. About 50% of these policies are intended to confront the Black maternal health crisis.^35^ Additionally, gray literature search will be conducted from research and committee reports, government reports, conference papers, White Ribbon Respectful Maternity Care Repository, and Columbia Public Health website.

In consultation with the medical librarian at the University of California San Francisco for their expertise, C.Y.O.-P. has generated a search strategy using medical subject headings on the terms “Maternity care providers,” “Perspective,” “Respectful maternity care,” “Person-centered maternity,” “Compassionate maternity care” together with BOOLEAN operators (“AND”/ “OR”) for PubMed, Embase, Web of Science, and CINAHL (see Supplementary Appendix A2). In addition, “Snowballing” through references identified in articles captured by the initial database search will be conducted.

### Study selection

The retrieved articles will be imported into Zotero reference management software and then duplicates will be removed. The remaining citations will be imported to Covidence and then additional duplicates will be removed. There will be two independent reviewers for this review. The lead reviewer will orient the other reviewer on the eligibility criteria and individual task assignments. The lead reviewer will conduct a title and abstract screening to obtain articles that meet the eligibility criteria. The reviewers will independently conduct the full-text screening for the selected articles. Identified studies that meet the eligibility criteria will be appraised for relevance and rigor by the reviewers. Reviewers must meet Cohen’s kappa inter-rater agreement of 0.61 and 0.80 for a substantial agreement. Also, we will resolve discrepancies through discussion and consultations with experts in the field. We will use the PRISMA flowchart to demonstrate the process of screening and identification of articles to be included in the scoping review, with reasons for exclusion documented.^36,37^

### Data charting, extraction, and management

Two independent reviewers will carry out the data extraction process. The researchers will extract data from included studies in the review using the standardized data extraction tool from Covidence to ensure the consistency of data extraction. The extracted data will consist of specific details about the populations, context, geographical location, study methods, provider perspectives and experiences of disrespect and abuse and obstetric violence in pregnancy and birthing, provider perspectives and experiences of respectful maternity care (RMC), as well as how providers incorporate the elements of RMC into their everyday practice.

The data will be collected using a standardized form that include domains such as study setting, sample characteristics, objectives, study design, data collection and analysis methods, study results, as well as the limitations of each individual study, and conclusions. Data extraction will also include identified themes and participant quotes for the qualitative studies. For the quantitative studies, the data extraction will involve data sources, predictors, outcome measures, and results. Both quantitative and qualitative reports will be considered in this review. Measures taken by quantitative studies to ensure validity and reliability will be assessed^38^; similar measures used by qualitative studies to ensure rigor or trustworthiness will also be evaluated.^39^ The review will assess the extracted data on providers’ perspectives on and experiences of disrespect, abuse, and obstetric violence during the pregnancy and postpartum period, facilitators and barriers to the provision of respectful care, and how providers observe the elements of RMC in their everyday practice. For missing data, authors will be contacted via phone or a total of three emails. If we do not get any information, that study will be excluded, and the reason will be documented.

### Risk of bias in individual studies

The risk of bias in individual studies will be assessed by two researchers for relevance and rigor using the Mixed Methods Appraisal Tool (MMAT).^40^ The MMAT is a quality assessment tool designed to assess qualitative, quantitative, randomized controlled trials, quantitative non-randomized, quantitative descriptive, and mixed methods studies. Since research on provider perspectives on RMC in the United States is still emerging, the characteristics and variables of the studies are heterogeneous. Therefore, the MMAT is an appropriate appraisal tool for the included studies. The MMAT consists of two screening questions that can be applied to all study designs, followed by five items unique to each study design.^40^ The screening questions will be used to determine if there are clear research questions and whether the data collected allows us to address the research question.^40^ Response options for all items are “Yes,” “No,” and “Can’t tell.”^40^ The methodological quality criteria for quantitative non-randomized trials, mixed methods, and qualitative designs will be used for this review. Quality assessment will be completed at the study level, and tables will be used to compare the qualities of the included studies, indicating for each study the risk of bias in each domain or component or item assessed and the overall study-level risk of bias. Justification will be provided for each judgment made.

### Data synthesis

The PRISMA-ScR will be used in this review.^33^ Based on the extent of heterogeneity of the included studies (in terms of study setting, sample characteristics, objectives, study design, data collection and analysis methods, study results), using STATA version 17 software, a random-effects meta-analysis will be conducted with the help of a statistician to obtain a pooled estimate value for providers’ perspectives on and experiences of disrespect, abuse, and obstetric violence during the pregnancy and postpartum period, facilitators and barriers to the provision of respectful care, and how providers observe the elements of RMC in their everyday practice. If quantitative synthesis is not appropriate due to the heterogeneity of the individual studies, a combination of narrative and thematic synthesis methodology will be employed. The descriptive narration will summarize the study’s methods, results, and conclusions. The emerging themes in the studies will be clustered according to themes as discussed by Lewin and colleagues in their study.^41^ The leading themes will be analyzed, and tabulation of the characteristics of the themes in prose will be included in the narrative.

### Ethics and dissemination

Ethics approval is not required for the scoping review. The results of this review will be submitted for open-access publication. The results will also be submitted as part of a doctoral qualifying examination and presented at conferences.

## Discussion

The findings from this scoping review will provide a comprehensive summary of clinicians’ perspectives on delivering facility-based maternity care that is free from discrimination and maltreatment, offering critical insights to inform policies and strategies aimed at improving the quality and equity of maternity care services. Centering health care providers’ experiences will reveal their understanding of respect, dignity, autonomy, and equity while also shedding light on the provider-level, facility-level, and system-level barriers that hinder the consistent delivery of RMC. These insights are particularly valuable given the inherent power imbalance between providers and birthing people,^42–44^ especially Black women, whose voices must remain central in efforts to improve maternity care. Juxtaposing clinicians’ perspectives with the lived experiences of birthing people offers a more holistic understanding of the challenges and gaps in providing dignified, person-centered care.

Furthermore, the review findings can guide the development of facility policies that utilize the RMC framework to establish evidence-based clinical guidelines and foster accountability. By identifying both obstacles and opportunities within clinical and institutional contexts, this study will not only reinforce the vital role of providers in ensuring RMC but will also lay the groundwork for future research, including investigations into the perspectives of hospital administrators and health care stakeholders. Ultimately, these contributions are crucial for shaping systemic reforms and creating maternity care environments where respect and equity are the standard rather than the exception.

## Abbreviations Used

CDC: Centers for Disease Control and Prevention
JBI: Joanna Briggs Institute
MMAT: Mixed Methods Appraisal Tool
PCC: Participants, Concepts, Context
PRISMA: Preferred Reporting Items for Systematic Review and Meta-analysis
PRISMA-ScR: Preferred Reporting Items for Systematic Review and Meta-analysis extension for Scoping Reviews
RMC: Respectful maternity care
SMM: Severe maternal morbidity
U.S.: United States
