# Supplementary material for: An Exploration of Maternal Health Care Providers’ Perspectives on Respectful Maternity Care in the United States: A Scoping Review Protocol
Source: Health Equity. 2025 Oct 1;9(1):612–7. doi: 10.1177/24731242251384093 (PMC12670658; doi:10.1177/24731242251384093)
Supplement: Supplementary Data [file 24731242251384093_suppl_datas1.docx]

**Title**

An Exploration of Maternal Healthcare Providers’ Perspectives on Respectful Maternity Care in the United States: A Scoping Review Protocol

**Corresponding Author**:

Celestine Yayra Ofori-Parku BSc, Dip.M, RM

PhD Candidate

Department of Family Health Care Nursing

School of Nursing, University of California San Francisco,

490 Illinois Street

San Francisco CA 94158

United States

[Celestine.Ofori-Parku@ucsf.edu](mailto:Celestine.Ofori-Parku@ucsf.edu)

**Appendix I**

|  | Inclusion criteria | Exclusion criteria |
| --- | --- | --- |
| Participants | Maternal healthcare providers: Physicians, nurses, APRNs, and midwives in the United States who work in a facility-based setting. | - Non-maternal healthcare providers, maternal healthcare providers, and midwives outside the U.S. - Maternal healthcare providers in non-facility-based settings. - Doulas, lactation consultants, and community health workers. |
| Concepts | - Provider perspectives and experiences of disrespect & abuse and obstetric violence in pregnancy and birthing - Provider perspectives and experiences of respectful maternity care (RMC). - Perspectives on the barriers to the provision of RMC - Perspectives on the facilitators to the provision of RMC - How providers incorporate the elements of RMC into their everyday practice - Article characteristics: year of publication, authors, author affiliations - Provider characteristics - Institutional constraints - Facility policies, practices, resources - Structural/systemic racism - Implicit bias training - Measures taken by individual studies to ensure rigor or trustworthiness of the studies | - Perspectives of birthing individuals. - Results that talk about the perspectives and experiences of non-providers - Results about perspectives and experiences providers outside the United States/healthcare settings. |
| Context | - Facility-based maternity care (hospitals, clinics, and birth centers) - Setting: United States - Time frame: Studies published between 2013-2024. - Language: Full text in English. - Peer-reviewed journals | Non-facility-based maternity care settings  Home birth |
| Type of Sources of Evidence Design | - Qualitative studies: Critical ethnography, grounded theory, case studies, interpretive phenomenology, comparative qualitative studies. Quantitative studies: Cross-sectional, case control, cohort studies, and intervention studies focusing on providers’ perspectives and experiences of (dis) respectful maternity care. | Editorials, commentary, viewpoints, protocols, practice guides, instrument development for women’s experience, and systematic or other reviews. |

### Appendix II: Search Strategy

**PubMed**

Updated March 5, 2025

| # | SEARCH | NUMBER OF PAPERS |
| --- | --- | --- |
| 1 | (Clinicians OR obstetricians OR healthcare providers OR maternity care providers OR doctors OR midwives OR nurses) | 6,069,891 |
| 2 | perspective OR perception OR experience OR knowledge | 3,243,166 |
| 3 | (Respectful maternity care OR Person-centered maternity care OR compassionate maternity care OR Humanization in birth OR mistreatment OR disrespectful Maternity care OR obstetric violence) | 99,452 |
| 4 | # 1AND #2 AND #3 | 13,002 |
| 5 | #4 AND Filters applied: in the past 10 years (2013- 2024) | 9,364 |
| 6 | #5 AND Filters applied: in the United States | 1,704 |
|  | #6 AND  Free full text, Full text, Comparative Study, Controlled Clinical Trial, Evaluation Study, Government Publication, Interview, Observational Study, Randomized Controlled Trial, Validation Study, English, from 2013/1/1 - 2024/12/31 | 108 |

**Embase**

Updated March 5, 2025

| # | SEARCH | NUMBER OF PAPERS |
| --- | --- | --- |
| 1 | (Clinicians OR obstetricians OR healthcare providers OR maternity care providers OR doctors OR midwives OR nurses) | 1,345.061 |
| 2 | perspective OR perception OR experience OR knowledge | 3,729,082 |
| 3 | (Respectful maternity care OR Person-centered maternity care OR compassionate maternity care OR Humanization in birth OR mistreatment OR disrespectful Maternity care OR obstetric violence) | 6,178 |
| 4 | #1 AND #2 AND #3 | 2,140 |
| 5 | #4 AND Filters applied: in the past 10 years (2013- 2024) | 1,737 |
| 6 | #5 AND Filters applied: in the United States | 474 |
| 7 | #6 AND ('case study'/de OR 'clinical article'/de OR 'clinical study'/de OR 'cohort analysis'/de OR 'comparative effectiveness'/de OR 'comparative study'/de OR 'controlled study'/de OR 'cross sectional study'/de OR 'delphi study'/de OR 'evidence based practice'/de OR 'exploratory research'/de OR 'feasibility study'/de OR 'grounded theory'/de OR 'human'/de OR 'interview'/de OR 'longitudinal study'/de OR 'major clinical study'/de OR 'observational study'/de OR 'open ended questionnaire'/de OR 'participatory research'/de OR 'pilot study'/de OR 'prospective study'/de OR 'qualitative research'/de OR 'quantitative study'/de OR 'questionnaire'/de OR 'retrospective study'/de OR 'semi structured interview'/de OR 'structured interview'/de OR 'structured questionnaire'/de) | 266 |

**Web of Science**

Updated March 5, 2025

| # | SEARCH | NUMBER OF PAPERS |
| --- | --- | --- |
| 1 | (Clinicians OR obstetricians OR healthcare providers OR maternity care providers OR doctors OR midwives OR nurses) | 1,357,654 |
| 2 | perspective OR perception OR experience OR knowledge | 7,459,980 |
| 3 | (Respectful maternity care OR Person-centered maternity care OR compassionate maternity care OR Humanization in birth OR mistreatment OR disrespectful Maternity care OR obstetric violence) | 7,303 |
| 4 | #1 AND #2 AND #3 | 1,334 |
| 5 | #4 AND Filters applied: in the past 10 years (2013- 2024) | 1,169 |
| 6 | #5 AND Filters applied: in the United States | 482 |
| 7 | #6 AND Filters applied: conference proceedings, article, early access, English. | 431 |

**CINAHL**

Updated March 5, 2025

| # | SEARCH | NUMBER OF PAPERS |
| --- | --- | --- |
| 1 | (Clinicians OR obstetricians OR healthcare providers OR maternity care providers OR doctors OR midwives OR nurses) | 926,768 |
| 2 | perspective OR perception OR experience OR knowledge | 1,016,865 |
| 3 | (Respectful maternity care OR Person-centered maternity care OR compassionate maternity care OR Humanization in birth OR mistreatment OR disrespectful Maternity care OR obstetric violence) | 1,980 |
| 4 | #1 AND #2 AND #3 | 400 |
| 5 | #4 AND Filters applied: in the past 10 years (2013- 2024) | 363 |
| 6 | #5 AND Filters applied: in the United States | 87 |
| 7 | #6 AND Filters applied: Academic journals and English language | 82 |
